# Infratentorial superficial siderosis: report of six cases and review of the literature

**DOI:** 10.3389/fnins.2024.1373358

**Published:** 2024-02-16

**Authors:** Lixia Deng, Yi Lin, Yu Lin, Weibin Huang

**Affiliations:** ^1^Department of Neurology, The First Affiliated Hospital of Fujian Medical University, Fuzhou, Fujian, China; ^2^Department of Neurology, The Third Hospital of Xiamen, Xiamen, Fujian, China; ^3^Fujian Institute of Neurology, The First Affiliated Hospital, Fujian Medical University, Fuzhou, Fujian, China; ^4^Department of Neurology, National Regional Medical Center, Binhai Campus of the First Affiliated Hospital, Fujian Medical University, Fuzhou, Fujian, China

**Keywords:** infratentorial superficial siderosis, magnetic resonance imaging, dural tear, deferiprone, T2-weighted imaging

## Abstract

**Objectives:**

To investigate the etiology, clinical manifestations, imaging features, and treatment of patients with infratentorial superficial siderosis (iSS), enhance clinicians' comprehension of this rare disease, and conduct oral deferiprone intervention and subsequent monitoring.

**Methods:**

Six patients diagnosed with iSS based on magnetic resonance imaging (MRI) and susceptibility weighted imaging (SWI) were enrolled from 2021 to 2023 at the First Affiliated Hospital of Fujian Medical University. Their clinical datas were summarized, and the etiology and imaging characteristics were analyzed. Follow-up was conducted through telephone or outpatient visits.

**Results:**

Among the 6 patients, there were 3 males and 3 females. The onset age ranged from 35 to 71 years, with an average onset age of 53 years. The clinical symptoms mainly included acoustic disturbances (6/6), gait imbalance (6/6), dysolfactory (6/6), cognitive impairment (2/6), epilepsy (2/6), and pyramidal tract sign (2/6). Evidence of superficial siderosis was observed on MRI across the cortex, brainstem, cerebellum, and spinal cord in all patients. T2-space sequence MRI revealed two instances of dural tear. During the follow-up period ranging from 1 month to 3 years, three patients who received oral deferiprone treatment showed improvement, whereas the remaining three patients who declined deferiprone treatment demonstrated progression.

**Conclusion:**

The primary clinical manifestations of iSS include bilateral sensorineural hearing disturbances, progressive cerebellar ataxia, and spinal cord lesions. The key diagnostic criteria involve the presence of linear hypointensity on T2-WI in the surface region of the nervous system. Dural tear caused by various factors is considered to be the most common cause of iSS, and its treatment mainly involves surgical intervention for hemorrhagic primary diseases as well as pharmacotherapy with deferiprone.

## 1 Introduction

The superficial siderosis of the central nervous system (SSCNS) is an uncommon neurodegenerative disorder resulting from chronic, recurrent or persistent leakage of minute quantities of erythrocytes into the subarachnoid space. SSCNS can be classified into cortical superficial siderosis (cSS) and infratentorial superficial siderosis (iSS), based on which brain regions are affected (Wilson et al., 2017). cSS primarily involves the cerebral cortex surface. Clinical manifestations include headache, focal nerve dysfunction, and cognitive impairment. iSS is defined as involvement of at least two sites within the brainstem, cerebellum or spinal cord with or without supratentorial distribution of hemosiderin deposition. The primary clinical manifestations of iSS encompass progressive cerebellar ataxia, sensorineural hearing loss in both ears, and spinal cord lesions. Additional prevalent neurological symptoms include cognitive impairment, epilepsy, hyposmia, dizziness, headache, etc. (Kumar, 2021).

In the past, the diagnosis of iSS primarily relied on postmortem examinations. Pathological analysis revealed deposits of hemosiderin, iron, and ferritin on the surface of the brainstem, cerebellum, and spinal cord, accompanied by secondary nerve cell degeneration, and demyelination changes. However, with the widespread utilization of magnetic resonance imaging (MRI) technology in recent years, an increasing number of cases of iSS have been identified. The presence of hypointense signal loops on the cortical surface, brainstem, cerebellum, and spinal cord can be observed in T2 sequences MRI as well as other iron-sensitive sequences in individuals with superficial siderosis.

Due to the limited number of cases, clinical heterogeneity, inadequate attention to etiological diagnosis, and lack of long-term follow-up data, iSS exhibits a high rate of missed diagnoses, low diagnostic rates, and insufficient treatment rates. It is crucial to accumulate relevant data from clinical and basic research promptly in order to facilitate the diagnosis and treatment of this disease. Therefore, the objective of this study was to prospectively enroll and analyze a cohort of six representative cases with iSS admitted to the First Affiliated Hospital of Fujian Medical University between 2021 and 2023. The study aimed to comprehensively characterize their clinical manifestations, including imaging findings, lumbar puncture results, audiological examinations, and other relevant outcomes. By combining these findings with existing literature evidence, we discussed its pathogenesis, potential etiology factors and treatment options. This comprehensive analysis aims to enhance our understanding and diagnostic capabilities regarding iSS while emphasizing the importance of exploring its underlying causes for developing accurate treatment strategies.

## 2 Materials and methods

### 2.1 Population

A total of six patients diagnosed with iSS were recruited from the First Affiliated Hospital of Fujian Medical University between 2021 and 2023. The inclusion criteria for patient selection included: (1) clinical admission with chief complaints of hearing loss, unsteady gait, or cognitive impairment; (2) Hypointense rings were observed on at least two locations within the brainstem, cerebellum, or spinal cord using T2 and other iron-sensitive sequences in MRI imaging.

### 2.2 Data collection

#### 2.2.1 Demographics

Two highly trained neurologists collected and sorted out the clinical data of 6 patients, including age, gender, age of onset, course of disease, clinical symptoms, and recorded the patient's signs, cognitive scores, imaging, electroencephalogram (EEG), cerebrospinal fluid (CSF) examination results, etc.

#### 2.2.2 Hearing evaluation

Hearing function was assessed using pure-tone audiometry. The hearing levels were represented by 3-frequency averages (3FA, 0.5/1/2 kilohertz, kHz) for both ears; 120 decibel hearing level (dBHL) values were substituted to calculate 3FA at frequencies where the hearing thresholds were not reached. Higher 3FA values represented worse hearing levels.

#### 2.2.3 Cognitive evaluation

Montreal Cognitive Assessment (MOCA): Cognitive function was assessed by MOCA (Beijing version), which includes visuospatial/executive function, naming, abstract/memory, attention/calculation, language, and orientation, is a rapid screening tool for mild cognitive impairment. It assesses a number of different cognitive domains. The total score of this scale is 30, and the normal value is ≥26.

#### 2.2.4 MRI

MR750 3.0 T superconducting magnetic resonance machine produced by Siemens Company of the Germany was used for MRI scanning. Routine T1-WI, T2-WI and SWI were performed in all six patients. T2-space sequence MRI were performed in two cases.

## 3 Results

### 3.1 General information

Among the 6 patients, there were an equal number of males and females (3 each), with ages ranging from 45 to 73 years and an average age of 59 years. The onset age ranged from 35 to 71 years, with an average onset age of 53 years. The duration of the disease varied between 1 and 10 years (Table 1).

**Table 1:** Clinical manifestation, cerebrospinal fluid, imaging, etiology, and treatment of the 6 patients with iSS.

case	sex	age (years)	symptoms	symptoms duration (years)	CSF RBC count (*10^9/L)	CSF protein (g/L)	siderosis on imaging	etiology	treatment
1	F	58	a, g, d, p	5	m/v	m/v	severe	dural tear	deferiprone
2	F	56	a, g, d, c	6	m/v	m/v	severe	inconcl.	deferiprone
3	F	67	a, g, d, c, e	1	2.4	0.47	moderate	inconcl.	heteropathy
4	M	73	a, g, d	2	m/v	m/v	moderate	inconcl.	heteropathy
5	M	55	a, g, d, e	5	0.1	0.71	severe	inconcl.	heteropathy
6	M	45	a, g, d, p	10	0.1	0.41	severe	dural tear	deferiprone

F, female; M, male; a, acoustic disturbances (tinnitus, hearing disturbances); g, gait imbalance; d, dysolfactory; c, cognitive impairment; e, epilepsy; p, pyramidal tract sign; CSF, cerebrospinal fluid; RBC, red blood cell; m/v, missing value; inconcl., inconclusive.

Siderosis on imaging was graded as mild (only infratentorial on SWI or T2-weighted images (WI), no or only thin rim on T2), moderate (supratentorial and infratentorial on SWI or T2-WI, thin rim on T2), severe (supratentorial and infratentorial on SWI or T2-WI, thick rim on T2).

### 3.2 Past medical history

None of the six iSS patients had a history of craniocerebral or lumbar trauma, nor did they undergo any related surgeries. Among them, two patients presented with diabetes and one patient exhibited hypertension as comorbidities.

### 3.3 Clinical symptoms

Among the 6 patients, all exhibited acoustic disturbances (6/6), gait imbalance (6/6), dysolfactory symptoms (6/6), while cognitive impairment, epilepsy, and pyramidal tract sign were present in 2 out of 6 patients each (Table 1).

### 3.4 Hearing and vestibular function examination

All six patients successfully completed the hearing function examination, revealing bilateral auditory impairment predominantly affecting high-frequency perception. Additionally, vestibular function assessment was conducted on all six cases, demonstrating a consistent weakness in semicircular canal response.

### 3.5 MRI

#### 3.5.1 Craniocerebral MRI

All six patients underwent brain MRI examination, and T2 low signal shadow around the cortex, cerebellum, brainstem, and craniocervical junction could be seen in all patients. All the 6 patients had cerebellar atrophy, especially cerebellar vermis.

#### 3.5.2 Spinal cord MRI

**Figure 1:**
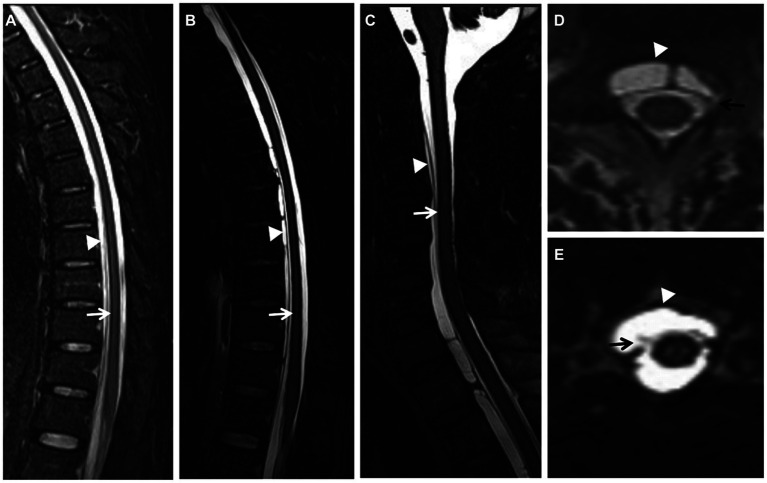
(Case 5) Extensive spinal superficial siderosis (SS) (**A–C**; arrows) and ventral epidural fluid collection at the cervical and thoracic levels (**A**–**E**; arrowheads). SS and ventral epidural fluid were more evident on T2-space sequences (**B**) than on T2-weighted images (WI) (**A**). Dural tear at the upper cervical level on axial T2-space sequence (**D**, **E**; arrows).

A complete spine MRI scan was performed on 6 patients, among whom superficial siderosis was observed in the cervical, thoracic, and lumbar medulla in 4 cases (severe), while it was observed in the cervical and thoracic medulla in 2 cases (moderate). Atrophy of the cervical pulp and thoracic pulp was evident in all 6 cases. T2-space sequence MRI revealed two instances of dural tear (Figure 1).

### 3.6 EEG

EEG was completed by three out of the six patients, all of whom exhibited a background activity characterized by diffuse slow-wave dominance.

### 3.7 CSF examination

Three out of the six patients underwent lumbar puncture and CSF examination. The CSF pressure was measured as 190, 105, and 90 mmH2O, respectively, (normal range: 80 ~ 180 mmH2O; conversion factor: 1 mmH2O = 0.0098 kPa). The total red blood cell counts were recorded as 2.4, 0.1, and 0.1 × 10^9/L, respectively, (normal range: 0). White blood cell counts were observed to be at levels of 6, 8, and 4 × 10^6/L, respectively, (normal range: up to a maximum of <8 × 10^6/L). CSF protein concentrations were measured as being at levels of 0.47, 0.71, and 0.41 g/L, respectively, (normal range: 0.15 ~ 0.45 g/L). Glucose and chloride levels remained within normal limits.

### 3.8 Follow-up observation

**Figure 2:**
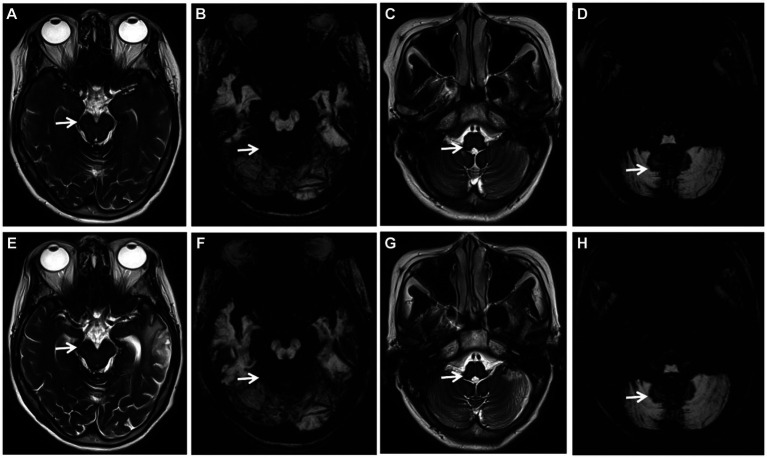
(Case 1) Axial T2-weighted images (WI) (**A**, **C**, **E**, **G**; arrows) and axial susceptibility-weighted imaging (SWI) (**B**, **D**, **F**, **H**; arrows) showing extensive SS, no progression of SS before treatment (**A**–**D**) and 2 years after deferiprone treatment (**E–H**).

After the diagnosis of iSS, patients were subjected to a follow-up period ranging from 1 month to 3 years, either through telephone or outpatient visits. The follow-up outcomes revealed that three patients who received oral deferiprone treatment showed improvement in cognitive function and gait balance, whereas the remaining three patients who declined deferiprone treatment demonstrated progression. The MRI examination conducted after a treatment duration of 2 years revealed no progression in SS (Figure 2).

## 4 Discussion

SSCNS is a rare central nervous system disorder characterized by the deposition of hemosiderin on the brain and spinal cord surfaces, leading to the development of neurological deficits. The prevalence of SSCNS is higher in the middle-aged and elderly population, with rates of 0.21% among individuals aged 50–69 years and 1.43% among those over 69 years (Pichler et al., 2017). Consistent with previous findings, four out of six patients in this study fell within the age range of 50–69 years.

The classification of iSS is further divided into classic and secondary types based on etiology (Wilson et al., 2017). Classic type iSS (ISS-1) is characterized by the absence of spontaneous or traumatic intracranial hemorrhage that could explain hemosiderin deposition. Conversely, secondary type iSS (ISS-2) refers to cases with a history of spontaneous or traumatic intracranial hemorrhage, such as aneurysm-induced spontaneous intracerebral hemorrhage, ventricular hemorrhage, or surgical trauma. In this study, all 6 patients were diagnosed with iSS-1 type, exhibiting involvement of at least two sites in the brainstem, cerebellum, or spinal cord; moreover, no instances of spontaneous or traumatic intracranial hemorrhage were observed that could account for the deposition of hemosiderin.

The clinical manifestations of iSS are dependent on the location and extent of hemosiderin deposition. In this study, all 6 cases involved the cortex, cerebellum, brainstem, I and VIII cranial nerves, and spinal cord. The corresponding clinical manifestations were cognitive impairment (2/6), epilepsy (2/6), gait imbalance (6/6), dysolfactory (6/6), acoustic disturbances (6/6), and pyramidal tract sign (2/6). Specifically: (1) Cognitive impairment and epilepsy: All six patients completed the MOCA assessment, with two demonstrating varying degrees of cognitive impairment primarily characterized by decreased recent memory, without a significant decline in visuospatial or executive function. This finding significantly diverges from previous studies that indicated cognitive impairment in iSS was predominantly associated with impaired executive function and visual memory (Jang et al., 2019; Chan et al., 2021; Fujii et al., 2023). However, these discrepancies may be attributed to the limited sample size and short follow-up duration. In future investigations, we aim to expand our sample size and extend the follow-up period. In this study, two patients presented with epilepsy; hemosiderin deposition in brain lobes such as the frontal subbasal surface, temporal lobe, and lateral fissure may serve as the pathological basis for seizures. Previous case reports have demonstrated that complete repair of cervical dura mater damage resulted in one patient with iSS not experiencing recurrent symptomatic epilepsy (Xu et al., 2021); (2) Gait imbalance: The main manifestations encompassed trunk ataxia and difficulty in walking straight; only one patient exhibited bilateral finger-nose test instability, suggesting that SSCNS ataxia may primarily involve a balance disorder. This finding is consistent with the study conducted by Weidauer et al. (2023). The cerebellar vermis may exhibit a higher susceptibility due to its abundant population of microglia and Bergmann glia, as well as its close proximity to the dorsal aspect of the fourth ventricle, which exposes it to an increased flow rate of cerebrospinal fluid; (3) Dysolfactory: the 6 patients in this study all complained of dysolfactory, and odor recognition tests all suggested different degrees of dysolfactory. Because the myelin sheath of the olfactory nerve is composed of glial cells, it is more vulnerable to damage (Kharytaniuk et al., 2022); (4) Acoustic disturbances: Pure tone hearing threshold tests were performed in all 6 patients in this study, suggesting binaural symmetric sensorineural hearing loss, and high-frequency hearing loss was more severe, which was consistent with the literature (Kumar, 2021). The VIII cranial nerve exits the brainstem through the pons and has a long period of immersion in the cerebrospinal fluid, and the myelin sheath is formed by glial cells, so it is most vulnerable to damage; (5) Pyramidal tract sign: In this study, 2 of 6 patients had pyramidal tract sign. iSS may be due to epidural hydrops caused by dural defects and the spinal cord is compressed, leading to myelopathy, mainly manifested as pyramidal tract sign, sensory disturbance and dysuria.

There are several potential sources of hemorrhage in SSCNS, including trauma, vascular malformation, central nervous system tumor, and cerebral amyloid angiopathy (CAA). CAA is the most common etiology of cSS in middle-aged and elderly patients (Charidimou et al., 2019a,b, 2020; Koemans et al., 2022) (Table 2). The source of hemorrhage in iSS-1 is generally unclear; however, dural tear has been identified as a common etiology (Takai and Taniguchi, 2021; Friedauer et al., 2023; Schievink et al., 2023) (Table 2). In this study cohort of six patients with iSS-1, two had confirmed dural tears. The pathophysiological of hemosiderin deposition in patients with dural defects is still unclear. However, previous studies have suggested the possible mechanism: CSF leaks into the epidural space through the ventral dural tear, and repetitive bleeding occurs from the epidural vessels that circulate back to the subarachnoid space through the dural tear, leading to hemosiderin deposition on the surface of the brain, and the spinal cord (Takai and Taniguchi, 2021; Yoshii et al., 2022).

**Table 2:** Differentiation between cSS and iSS.

Items	cSS	iSS
Classification of type	Focal type (≤3 sulci involvement); Disseminated type (>3 sulci involvement)	Type 1 (classical); Type 2 (secondary)
Most common cause	CAA	Dural tear
Clinical presentation	TFNEs, cognitive impairment	Progressive hearing loss, ataxia and myelopathy
Imaging on MRI	Linear hypointensity on T2-WI was restricted to the supratentorial	Linear hypointensity on T2-WI involved at least two sites (brain stem, cerebellum, spinal cord), with or without supratentorial
CSF studies	Red cells	Red cells
Treatment	Surgical removal of the source of bleeding; deferiprone	Surgical removal of the source of bleeding; deferiprone
Differential diagnosis	TIA, migraine with aura, epilepsy, et al.	Spinocerebellar ataxia, et al.

cSS, cortical superficial siderosis; iSS, infratentorial superficial siderosis; CAA, cerebral amyloid angiopathy; TFNEs, transient focal neurologic symptoms; TIA, transient ischemic attack.

With the continuous advancement of neuroimaging techniques, MRI of the brain and spinal cord has emerged as a crucial diagnostic tool for iSS. iSS is characterized by a distinct low T2 linear signal on the surface of the central nervous system in contact with CSF, while SWI exhibits higher sensitivity toward hemosiderin deposition (Stabile et al., 2018; Wagner et al., 2019). In this study, all six patients underwent comprehensive cranial MRI examinations which revealed T2 hypointense shadows surrounding the cortex, cerebellum, brainstem, and craniocervical junction. Complete MRI evaluations of the spinal cord in six cases demonstrated iron deposition signals on its surface along with fluid accumulation within the spinal canal; additionally, two cases showed dural tears using T2-space sequences (Figure 1). Our findings indicate that T2-space sequences are superior to conventional T2 sequences in identifying SS and dural tear due to their enhanced contrast between the dura and CSF. Dural tear represents the most common etiology for iSS; however, fluid accumulation within the spinal canal merely suggests potential cerebrospinal fluid leakage without providing information regarding its exact location. Complementary techniques such as CT myelography, digital subtraction myelography (DSM), or magnetic resonance myelography can be employed to identify fistulas based on institutional experience. Therefore, an initial routine MRI scan encompassing the entire central nervous system should be performed prior to selecting an appropriate imaging modality for fistula detection.

EEG was completed by three out of the six patients, all of whom exhibited a background activity characterized by diffuse slow-wave dominance. The three patients with iSS exhibited extensive cortical superficial siderosis, leading to cortical atrophy, which was characterized by the presence of diffuse slow-wave dominance on the EEG. In future investigations, we will strive to augment the sample size and continuously monitor the EEG performance.

In this study, the average time from onset to diagnosis of iSS was 1–10 years, and the average diagnosis time was about 5 years. We hypothesize that the prolonged interval between symptom onset and iSS diagnosis may be attributed to the following factors: (1) insufficient clinical understanding of the disease, lack of unified diagnostic criteria; (2) some asymptomatic patients with recurrent micro cerebral hemorrhage did not enter the medical procedure; (3) the early symptoms of the disease are mild, and the first MRI examination rate and follow-up rate are not high; (4) there are large inter-individual differences in the absorption of heme iron in brain tissue.

Up to now, there is no recognized and definite treatment for SSCNS, mainly including surgery, drug therapy and other symptomatic treatments: (1) Surgical treatment is suitable for patients with definite hemorrhagic lesions, dural abnormalities, aneurysms, vascular malformations or central nervous system tumors. Among the reported cases, a minority of patients have undergone surgical intervention, and its long-term clinical efficacy remains uncertain (Takai and Taniguchi, 2021; Yoshii et al., 2022); (2) Deferiprone is one of the main treatment methods at present. In literature reports, low-dose deferiprone has a certain curative effect (Kessler et al., 2018; Cossu et al., 2019; Flores Martin et al., 2021), but Sammaraiee et al. (2020) have raised doubts about the safety and tolerance of long-term use of deferiprone treatment in patients with iSS, 40% of the cases withdrew from treatment due to granulocytopenic sepsis and fatigue side effects, of which the largest proportion was granulocytopenic sepsis (30%). Therefore, it is necessary to further study the risk of long-term treatment in the future; (3) Other symptomatic treatments: for patients with hearing loss, the suitableness of cochlear implantation can be evaluated. Although hearing loss is caused by post-cochlear lesions, some patients have different degrees of benefits after cochlear implantation (Artukarslan et al., 2022). In this study, 2 of the 6 patients with iSS were confirmed to have a dural tear, but both refused surgery and received deferiprone treatment, while the other patient without a dural tear was also treated with deferiprone. Three patients who received deferiprone treatment were followed up for 1 month to 3 years, and all showed improvement in gait balance, but no improvement in hearing and olfactory dysfunction. The MRI examination conducted after a treatment duration of 2 years revealed no progression in SS (Figure 2). No side effects were observed in these three patients. Three of the six patients received cochlear implantation, and their hearing was partially improved after surgery, and their quality of life was improved. There are few cases of iSS treated with deferiprone and cochlear implantation in China, and there is a lack of long-term follow-up studies. We will continue to follow up patients to observe the long-term efficacy.

This study has a small sample size and is a single-center study. A prospective international centralized register of patients should be developed to inform the design and conduct of a multicentre, placebo-controlled, randomized clinical trial to unify the diagnostic criteria for iSS, and evaluate the efficacy of deferiprone. Future research should prioritize the following aspects: (1) conducting prospective longitudinal studies to evaluate the therapeutic effects of deferiprone and dural tear repair; (2) investigating the role of biomarkers, such as CSF ferritin; (3) elucidating the precise mechanism underlying chronic erythrocyte leakage in patients with dural tears; and (4) further clarifying the mechanisms responsible for hemosiderin retention and neuronal damage resulting from chronic subarachnoid hemorrhage.

## 5 Conclusion

iSS is a disease characterized by the deposition of hemosiderin on the surface of the brain, spinal cord and cranial nerves, resulting in sensorineural hearing loss, cerebellar ataxia and pyramidal signs. The linear hypointensity on T2-WI is the main basis for the diagnosis of iSS. The most prevalent etiology of iSS is dural tear resulting from various causes, and the management of this condition primarily encompasses surgical intervention for hemorrhagic primary diseases and pharmacotherapy with deferiprone.

## Data availability statement

The original contributions presented in the study are included in the article/supplementary material, further inquiries can be directed to the corresponding author.

## Ethics statement

The studies involving humans were approved by the ethics committee of the First Affiliated Hospital of Fujian Medical University ([2021]462). The studies were conducted in accordance with the local legislation and institutional requirements. The participants provided their written informed consent to participate in this study. Written informed consent was obtained from the individual(s) for the publication of any potentially identifiable images or data included in this article.

## Author contributions

LD: Data curation, Formal analysis, Investigation, Methodology, Validation, Visualization, Writing – original draft, Writing – review & editing. YiL: Data curation, Investigation, Resources, Supervision, Writing – review & editing. YuL: Data curation, Investigation, Visualization, Writing – review & editing. WH: Data curation, Funding acquisition, Investigation, Supervision, Visualization, Writing – original draft, Writing – review & editing.
