# Corrigendum: Infratentorial superficial siderosis: report of six cases and review of the literature

**DOI:** 10.3389/fnins.2024.1388356

**Published:** 2024-03-06

**Authors:** Lixia Deng, Yi Lin, Yu Lin, Weibin Huang

**Affiliations:** ^1^Department of Neurology, The First Affiliated Hospital of Fujian Medical University, Fuzhou, Fujian, China; ^2^Department of Neurology, The Third Hospital of Xiamen, Xiamen, Fujian, China; ^3^Fujian Institute of Neurology, The First Affiliated Hospital, Fujian Medical University, Fuzhou, Fujian, China; ^4^Department of Neurology, National Regional Medical Center, Binhai Campus of the First Affiliated Hospital, Fujian Medical University, Fuzhou, Fujian, China

**Keywords:** infratentorial superficial siderosis, magnetic resonance imaging, dural tear, deferiprone, T2-weighted imaging

In the published article, there was an error regarding the affiliations for Yi Lin, Yu Lin, and Weibin Huang. As well as having affiliations 1 and 3, they should also have *Department of Neurology, National Regional Medical Center, Binhai Campus of the First Affiliated Hospital, Fujian Medical University, Fuzhou, Fujian, China*.

The authors apologize for this error and state that this does not change the scientific conclusions of the article in any way. The original article has been updated.

